# Vesicle biomechanics in a time-varying magnetic field

**DOI:** 10.1186/s13628-014-0016-0

**Published:** 2015-01-21

**Authors:** Hui Ye, Austen Curcuru

**Affiliations:** Department of Biology, Loyola University Chicago, 1032 W. Sheridan Rd, Chicago, IL 60660 USA; Departments of Physics, Loyola University Chicago, 1032 W. Sheridan Rd, Chicago, IL 60660 USA

**Keywords:** Time varying magnetic field, Vesicle, Biomechanics, Modeling, Transcranial magnetic stimulation (TMS)

## Abstract

**Background:**

Cells exhibit distortion when exposed to a strong electric field, suggesting that the field imposes control over cellular biomechanics. Closed pure lipid bilayer membranes (vesicles) have been widely used for the experimental and theoretical studies of cellular biomechanics under this electrodeformation. An alternative method used to generate an electric field is by electromagnetic induction with a time-varying magnetic field. References reporting the magnetic control of cellular mechanics have recently emerged. However, theoretical analysis of the cellular mechanics under a time-varying magnetic field is inadequate.

We developed an analytical theory to investigate the biomechanics of a modeled vesicle under a time-varying magnetic field. Following previous publications and to simplify the calculation, this model treated the inner and suspending media as lossy dielectrics, the membrane thickness set at zero, and the electric resistance of the membrane assumed to be negligible. This work provided the first analytical solutions for the surface charges, electric field, radial pressure, overall translational forces, and rotational torques introduced on a vesicle by the time-varying magnetic field. Frequency responses of these measures were analyzed, particularly the frequency used clinically by transcranial magnetic stimulation (TMS).

**Results:**

The induced surface charges interacted with the electric field to produce a biomechanical impact upon the vesicle. The distribution of the induced surface charges depended on the orientation of the coil and field frequency. The densities of these charges were trivial at low frequency ranges, but significant at high frequency ranges. The direction of the radial force on the vesicle was dependent on the conductivity ratio between the vesicle and the medium. At relatively low frequencies (<200 KHz), including the frequency used in TMS, the computed radial pressure and translational forces on the vesicle were both negligible.

**Conclusions:**

This work provides an analytical framework and insight into factors affecting cellular biomechanics under a time-varying magnetic field. Biological effects of clinical TMS are not likely to occur via alteration of the biomechanics of brain cells.

## Background

Cellular physiology is modified in the interaction between cells and an electric field. Cells receive mechanical signals that activate a biochemical cascade of events and produce various biological responses. Evidence of the effects of an electric field on cellular mechanics is abundant in the literature. As reported, cell membrane could be deformed inside an electric field [1,2]. Cell elongation perpendicular to the electric field has also been observed in human adipose tissue-derived stem cells [3]. An external electric field could generate undulation on a poorly conductive membrane [4]. Finally, electric fields generated by a microelectrode could induce stress in the cell membranes, leading to tension and poration [5].

To investigate the mechanisms underlying field-induced biomechanics on the cell membrane, closed pure lipid bilayer membranes (vesicles) were used for electrodeformation experimentation [6] [7]. Vesicles exposed to a direct-current (DC) electric pulse could be deformed into elliptical [8] or cylindrical shapes [7]. Recent interests included the use of a substantially strong DC field to induce vesicle deformation [9]. Paralleled with experimental approaches, theoretical works have also been proposed to quantitatively reveal the biomechanical mechanisms of membrane deformation under these conditions. For example, Grosse and Schwan [10] resolved the membrane potential for a spherical cell when an electric field has been imposed. Hyuga et al. [11] used Maxwell stress tensor to calculate the normal component of the force on the cell membrane in a DC field. Their model considered the dynamic deformation of the vesicles by assuming a permeable, conducting membrane. Others [6] modeled the effects of alternating current (AC) electric field on the vesicle, and investigated the impact of field frequency on cell deformation. Considering the balance of electric, hydrodynamic bending, and tension stresses exerted on the cell membrane, the vesicle showed various frequency – dependent kinematic changes in the AC electric field including deformation, orientation, translation (dielectrophoresis), and rotation [12]. Orientation of the cells can be predicated by the calculated torque on the cell, and this method has been used to explain the electro-orientation of erythrocytes [13]. Collectively, these works share insight about the forces involved in deforming the membrane, which rely primarily on the interaction between the electric field and the free charges (ions) that accumulate on the membrane surface of the cell.

Magnetic fields have also been shown to affect cellular mechanics. Previous investigations have reported a myriad of responses in support of magnetic field effects on cellular dynamics. For example, pulsed electromagnetic fields caused long-term morphological changes in cultured human chondrocytes [14]. In one study, a force between the action currents in a nerve and the static MRI magnetic field caused the nerve to move [15], while in another, motion induced by a magnetic field on a magnetic particles or magnetizable material (magnetophoresis) was used for the isolation of blood cells [16] and extracellular vesicles [17]. However, the most prevailing usage of the field in clinical treatment is the transcranial magnetic stimulation (TMS), in which an electric field is induced inside the brain for the treatment of depression [18], seizures [19,20], Parkinson’s disease [21], and Alzheimer’s disease [22]. Literature regarding the magnetic field’s biomechanical effects on single cells is limited. A theoretical analysis of time-varying magnetic field on cellular mechanics could provide insights into the possibility of magnetic control of cellular physiology. In the context of TMS practice, it is unknown if the parameters implemented by TMS would cause membrane deformation or any biomechanical alterations.

We have recently studied the excitability of the cells by computing transmembrane potential on a spherical soma [23], an axon [24], and internal organelle such as a mitochondrion [25] under the stimulation of a time varying magnetic field. In this paper, we computed the pressures, forces, and torques generated by a time-varying magnetic field on a simple vesicle model, and estimated the extent to which magnetic fields used in TMS practice could affect these measurements.

## Methods

### Spherical vesicle model in a low frequency magnetic field

**Figure 1:**
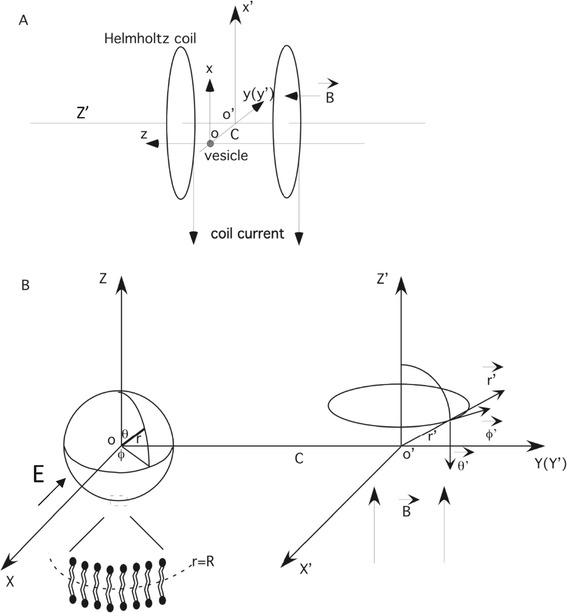
**A vesicle model under a spatially uniform, time-varying magnetic field. A**. A magnetic field generated by a Helmholtz coil pair and the location of the targeted vesicle. The current flowing in the coil generated a sinusoidally alternating magnetic field, which in turn induced an electric field around the vesicle, along the x-axis. The small sphere represented a vesicle that centers at point O, a point close to the center axis of the Helmholtz coil pair (O’Z’). The distance between the center of the vesicle (O) and the common axis (O’Z’) of the Helmholtz coil pair was C. **B**. Transverse view of the model showing a simple sphere that represented the vesicle in a spherical coordinates (*r*, *θ*, *φ*). The externally applied magnetic field was in spherical coordinates (*r* ', *θ* ', *φ* '). The zoomed region illustrated the lipid bilayer structure of the membrane. The induced electric field (E) oscillated along the x-axis.

Figure 1 shows the basic geometry of the modeled vesicle located between a pair of a Helmholtz coils. Two coordinate systems were utilized to represent the vesicle and the coil, respectively. The spherical vesicle was represented in a spherical coordinate system (*r*, *θ*, *ϕ*), centered at point *O*. The dielectric permittivities and conductivities inside and outside the vesicle were *ε*_*i*_, *ε*_*o*_ and *σ*_*i*_, *σ*_*o*_, respectively. The vesicle had a radius of *R*. The bilayer thickness was appropriately 5 nm, thus on the length scale of a cell-size vesicle (radius ~ 10 μm), the bilayer membrane can be regarded as a two-dimensional surface. As in the literature [9,26], the thickness of the membrane was considered to be zero, and the electric resistance of the membrane was assumed to be negligible.

The magnetic field was represented in a cylindrical coordinate system (*r* ', *ϕ* ', *z* '), with the axis of the coils overlapped with the OZ’ axis. The distance between the center of the cell (*O*) and the axis of the coil (*O* ') was *C*. The externally applied, sinusoidally alternating magnetic field was symmetric about the *O* ' *Z* ' axis in this coordinate system. Mathematically, the magnetic field could be represented as $\overset{\rightharpoonup }{B}=\overset{\rightharpoonup }{Z}\hbox{'}{B}_0{e}^{j\omega t}$, where $\overset{\rightharpoonup }{Z}\hbox{'}$ the unit vector in the direction of *O* ' *Z* ' and ω the angular frequency of the magnetic field, and $j=\sqrt{-1}$ the imaginary unit.

### Governing equations for electrodynamics problems

A time-varying magnetic field induces an electric field. Assuming the presence of the model vesicle did not affect the distribution of the induced field, we used Faraday’s law to compute the induced electric field:

1 $$ {\displaystyle \oint \overset{\rightharpoonup }{E}\cdot d\overset{\rightharpoonup }{l}=-{\displaystyle \iint \frac{\partial \overset{\rightharpoonup }{B}}{\partial t}}}\cdot d\overset{\rightharpoonup }{A} $$

E was in the *φ* ' direction in the cylindrical coordinates (*r* ', *ϕ* ', *z* '). By integration,

$$ 2\pi CE=-j\omega {B}_0\pi {C}^2 $$,

we had the intensity of the induced electric field

2 $$ E=\frac{-j\omega {B}_0C}{2} $$

For the two representative field frequencies that were analyzed in this paper (10 KHz and 200 KHz), the induced electric field was 120 V/m (C = 1 cm) at point O for 10 KHz (TMS). At this field intensity, cell migration has been observed [27]. In order to cause vesicle deformation, an electric field would be as large as 240000 V/m [7].

### Laplace equation

The presence of the vesicle in the space introduced free charge distribution. The electric field induced by the time-varying magnetic field around the vesicle was

3 $$ \overrightarrow{E}=-j\omega \overrightarrow{A}-\nabla V $$

where $\overset{\rightharpoonup }{A}$ was the magnetic vector potential induced by the current in the coil. The potential *V* was the electric scalar potential due to charge accumulation that appears from the application of a time-varying magnetic field. In spherical coordinates (*r*, *θ*, *ϕ*), $\nabla V=\left(\frac{\partial V}{\partial r},\frac{1}{r}\frac{\partial V}{\partial \theta },\frac{1}{r \sin \theta}\frac{\partial V}{\partial \phi}\right)$. For low frequency stimulation, we used quasi-static approximations. In charge-free regions, *V* was obtained by solving Laplace’s equation

4 $$ {\nabla}^2V=0 $$

### Magnetic vector potential $\overrightarrow{A}$ in spherical coordinates (*r*, *θ*, *ϕ*)

When the center of the magnetic field was at point *O* ', $\overset{\rightharpoonup }{B}$ was in the direction of $\overset{\rightharpoonup }{Z}\hbox{'}$. This is because

5 $$ \overset{\rightharpoonup }{B}=\nabla \times \overset{\rightharpoonup }{A} $$,

where vector potential $\overset{\rightharpoonup }{A}$ was in the direction of $\overset{\rightharpoonup }{\phi}\hbox{'}$ (Figure 1). In cylindrical coordinates (*r* ', *ϕ* ', *z* '), the magnetic vector potential was expressed as (Appendix A):

6 $$ \overset{\rightharpoonup }{A}\hbox{'}=-\frac{r\hbox{'}{B}_0}{2}{e}^{j\omega t}\overset{\rightharpoonup }{\phi}\hbox{'} $$

In order to calculate the potential distribution in the model vesicle, one needs to have an expression for $\overset{\rightharpoonup }{A}$ in spherical coordinates (*r*, *θ*, *ϕ*). By coordinate transformation (Appendix B), we obtained the magnetic vector potential $\overset{\rightharpoonup }{A}$ in spherical coordinates (*r*, *θ*, *ϕ*):

7 $$ \overset{\rightharpoonup }{A}=\overset{\rightharpoonup }{r}{A}_{or}+\overset{\rightharpoonup }{\theta }{A}_{o\theta }+\overset{\rightharpoonup }{\phi }{A}_{o\varphi } $$

The vector potential components in the $\overset{\rightharpoonup }{r},\overset{\rightharpoonup }{\theta },\overset{\rightharpoonup }{\phi }$ directions were:

8 $$ {A}_{or}=\frac{B_0}{2}C \sin \theta \cos \phi $$

9 $$ {A}_{o\theta }=\frac{B_0}{2}C \cos \theta \cos \phi $$

10 $$ {A}_{o\phi }=\frac{B_0}{2}\left(r \sin \theta -C \sin \phi \right) $$

### Induced surface charges

At the boundary that separates the media with different electrical properties, free charges presented and caused a discontinuity in the normal component of the displacement vector.

11 $$ {\rho}_s=\overset{\rightharpoonup }{n}\bullet \left({\varepsilon}_o{\overset{\rightharpoonup }{E}}_o-{\varepsilon}_i{\overset{\rightharpoonup }{E}}_i\right)\;\mathrm{at}\;r=R $$

where $\overset{\rightharpoonup }{n}$ denoted outward unit normal vector and *ρ*_*s*_ charge density.

### Boundary conditions

Four boundary conditions were considered in the derivation of the potentials induced by the time-varying magnetic field. 1. The potential was continuous across the boundary of two different media. ***V***_***o***_ = ***V***_***i***_ at ***r*** = ***R*** as in the previous works [9,26]. 2. Conservation of electric current at the interface requires the normal component of the current density to be continuous across two different media. For materials such as pure conductors, it is equal to the product of the electric field and the conductivity of the media. During time-varying field stimulation, the “complex conductivity” (defined as *S* = *σ* + *jωε*) was used to account for the dielectric permittivity of the material [28-30]. Here, *σ* was the conductivity, *ε* was the permittivity of the tissue, and *ω* was the angular frequency of the field. Therefore, on the vesicle/medium interface

12 $$ {S}_o{E}_{or}={S}_i{E}_{ir} $$

where *S*_*o*_ = *σ*_*o*_ + *jωε*_*o*_ and *S*_*i*_ = *σ*_*i*_ + *jωε*_*i*_ were the complex conductivities. 3. The electric field at an infinite distance from the cell was not perturbed by the presence of the vesicle, and 4. The electric potential inside the cell (*r* = 0) was finite.

### Force and torques generated on the vesicle

The force generated on the vesicle was a result of the interaction between the free charges and electric field. The electric traction on the vesicle surface had radial and tangential components. The tangential force generated the rotational torque.

### Model parameter and simulation

Table 1 lists the parameters used for the model. To quantitatively investigate the amount of forces and torques generated on the vesicle, we chose their geometrical and electrical parameters (standard values, the lower and upper limits) from the literature [29]. The frequency range of interest was determined to be between 2 kHz - 200 kHz. The upper limit was determined by calculating the reciprocal value of the rising phase of a current pulse during peripheral nerve stimulation [31,32]. Most frequencies used in the experimental practices were lower than this value [33]. The intensity and frequency parameters were picked to represent a brain neuron under transcranial magnetic stimulation (TMS), with the field intensity of 2 Tesla. The standard frequency of the magnetic field was estimated to be 10 kHz, as the rising time of single pulses was ~100 *μs* during TMS. This yielded the peak value of *dB*/*dt* = 2 × 10^4^*T*/*s*.

**Table 1:** **Model parameters**

**Parameters**	**Standard value**	**Lower limit**	**Upper limit**
Medium conductivity (*σ*_*o*_, S/m)	0.3 (1)	0.01 (1)	1.2 (1)
Cytoplasmic conductivity (*σ*_*o*_, S/m)	0.3 (1)	0.2 (1)	1.2 (1)
Vesicle radius (*R*, *μm*)	10 (2)	5 (2)	100 (2)
Magnetic field frequency (*f*, *kHz*)	10	2	200 (3,4)
Coil axis - cell distance (*C*, *cm*)	1	0	10
Medium dielectric permittivity (*ε*_*o*_, As/Vm)	6.4 × 10^− 10^ (2)	-	-
Cytoplasmic dielectric permittivity (*ε*_*o*_, As/Vm)	6.4 × 10^− 10^ (2)	-	-
Magnetic field intensity (B_0_, Tesla)	2	-	-

1 In [28].

2 In [29].

3 In [32].

4 In [31].

## Results

### Electric field induced by the time-varying magnetic field

In spherical coordinates (*r*, *θ*, *ϕ*), the solution for Laplace’s equation (4) was written as

13 $$ {V}_n=\left({C}_nr+{D}_n\frac{1}{r^2}\right) \sin \theta \cos \phi $$

where *C*_*n*_, *D*_*n*_ were unknown coefficients (n = o and i). These coefficients were solved with the given boundary conditions (Appendix C).

14 $$ {V}_o=\frac{j\omega {B}_0C}{2}\frac{R^3}{r^2}\frac{S_o-{S}_i}{2{S}_o+{S}_i} \sin \theta \cos \varphi $$

15 $$ {V}_i=\frac{j\omega {B}_0C}{2}\frac{S_o-{S}_i}{2{S}_o+{S}_i}r \sin \theta \cos \varphi $$

Using equation (3), we obtained the expression of the magnetically-induced electric field around the vesicle

16 $$ \begin{array}{l}{E}_{or}=-\frac{j\omega {B}_0C}{2}\left(1+\frac{2{R}^3}{r^3}\frac{S_i-{S}_o}{2{S}_o+{S}_i}\right) \sin \theta \cos \varphi \\ {}=-\frac{j\omega {B}_0C}{2}\frac{3{S}_i}{2{S}_o+{S}_i} \sin \theta \cos \varphi \end{array} $$

(when r = R)

17 $$ \begin{array}{l}{E}_{o\theta }=-\frac{j\omega {B}_0C}{2}\left(1-\frac{R^3}{r^3}\frac{S_i-{S}_o}{2{S}_o+{S}_i}\right) \cos \theta \cos \varphi =\\ {}-\frac{j\omega {B}_0C}{2}\frac{3{S}_o}{2{S}_o+{S}_i} \cos \theta \cos \varphi \end{array} $$

(when r = R)

18 $$ \begin{array}{l}{E}_{o\varphi }=\frac{j\omega {B}_0}{2}\left[C\left(1-\frac{R^3}{r^3}\frac{S_i-{S}_o}{2{S}_o+{S}_i}\right) \sin \varphi -r \sin \theta \right]\\ {}=\frac{j\omega {B}_0}{2}\left[C\frac{3{S}_o}{2{S}_o+{S}_i} \sin \varphi -R \sin \theta \right]\end{array} $$

(when r = R).

Electric field distribution inside a vesicle was

19 $$ {E}_{ir}=-\frac{j\omega {B}_0C}{2}\frac{3{S}_o}{2{S}_o+{S}_i} \sin \theta \cos \varphi $$

20 $$ {E}_{i\theta }=-\frac{j\omega {B}_0C}{2}\frac{3{S}_o}{2{S}_o+{S}_i} \cos \theta \cos \varphi $$

21 $$ {E}_{i\varphi }=\frac{j\omega {B}_0}{2}\left(C\frac{3{S}_o}{2{S}_o+{S}_i} \sin \varphi -r \sin \theta \right) $$

### Surface charge induced by the time-varying magnetic field

Surface charge density on the vesicle was

22 $$ {\rho}_s\left(R,\theta, \varphi \right)={\varepsilon}_o{E}_{or}-{\varepsilon}_i{E}_{ir}=-\frac{j\omega {B}_0C}{2}\frac{3\left({S}_i{\varepsilon}_o-{S}_o{\varepsilon}_i\right)}{2{S}_o+{S}_i} \sin \theta \cos \varphi $$

**Figure 2:**
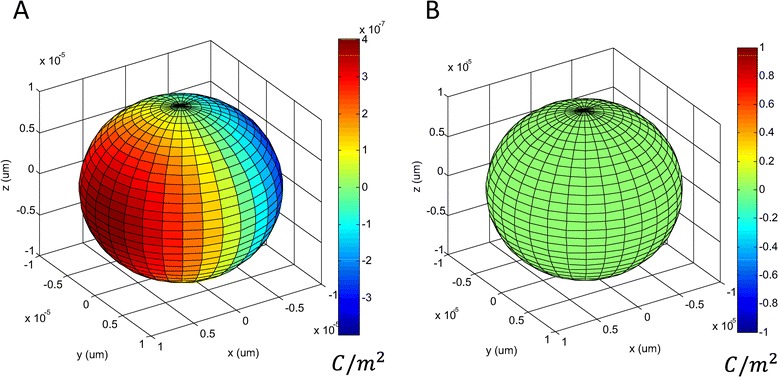
**Surface charge distribution induced by the time-varying magnetic field. A**. The plot demonstrated an instant pattern of surface charge distribution. The orientation of the vesicle to the coil was the same as that shown in Figure 1B. The color represented the amount of the charge density (C/m^2^) calculated with the standard parameters in Table 1. Field frequency was 10 KHz. *σ*_*o*_ = 1.2*S*/*m. σ*_*i*_ = 0.3*S*/*m*. **B**. There was no accumulation of surface charges if the two media were set to be electrically identical (*σ*_*o*_ = *σ*_*i*_ and *ε*_*o*_ = *ε*_*i*_).

**Figure 3:**
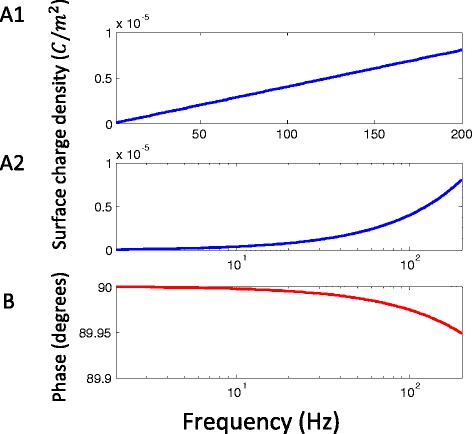
**Frequency dependence of surface charges on the vesicle under the time-varying magnetic field.** The figure plotted the absolute value of the amplitude of the surface charge density at the location (*π*/2, 0). Notation and parameter values were the same as in Figure 2A. **A**. Maximal amplitude of surface charges *ρ*_*s*_ as a function of the field frequency in a linear plot (A1) and in a log plot (A2). **B**. Phase of *ρ*_*s*_ as a function of the field frequency. Phase lag was defined between the phases of the magnetic field and *ρ*_*s*_.

The density of the induced electric charges (*ρ*_*s*_) was independent of cell size (R), but was dependent on the dielectric properties of the cell’s vicinity and its relative orientation to the magnetic coil (Figure 2A). The surface charges only accumulated on the boundary of the two inhomogeneous media (Figure 2B). We computed the maximal intensity of the surface charges with parameters provided in Table 1, and the frequency dependency of surface charges was illustrated in Figure 3. An increase in field frequency caused an increase in surface charges (Figure 3A1 and A2), and a decrease in the phase (Figure 3B). These changes were more prominent when field frequency exceeded 100 K Hz. At f = 10 kHz, the TEM frequency, the induced surface charge density was *ρ*_*s*_ = 4.0 × 10^− 7^*C*/*m*^2^, and at f = 200 kHz, the induced surface charge density was *ρ*_*s*_ = 8.0 × 10^− 6^*C*/*m*^2^. Induced charge density was significantly smaller than the intrinsic charges carried by the proteins on the cell under physiological conditions [34].

The net induced charge on the vesicle was

23 $$ {Q}_s={\displaystyle \underset{\theta, \varphi }{\iint }{\rho}_sda={\displaystyle \underset{\theta, \varphi }{\iint }{\rho}_s{R}^2 \sin \theta d\theta d\varphi =0}} $$

It yielded a value of zero because the induced charges could not leave the vesicle surface [35]. Here, *dȃ* = *R*^2^ sin *θdθdφȓ* was a surface element in the *ȓ* direction.

### Radial pressure due to interactions between the magnetically-induced electric field and the induced charges *ρ*_*s*_

We next analyzed the surface biomechanics of the spherical vesicle including: pressure for pulling and compression, forces for vesicle translation, and torques for rotation.

We first calculated the pressure on the *ȓ* direction that could compress or expand the vesicle surface. The force generated on a surface charge equals the product of the charge and the average of the electric field on both sides of the surface [36]. Pressure (force per unit area) on the vesicle surface was:

24 $$ {P}_r=\frac{1}{2}\left({E}_{or}+{E}_{ir}\right){\rho}_s=-\frac{9}{8}{\omega}^2{B}_0^2{C}^2\frac{\left({S}_o+{S}_i\right)\left({S}_i{\varepsilon}_o-{S}_o{\varepsilon}_i\right)}{{\left(2{S}_o+{S}_i\right)}^2}{ \sin}^2\theta { \cos}^2\varphi $$

**Figure 4:**
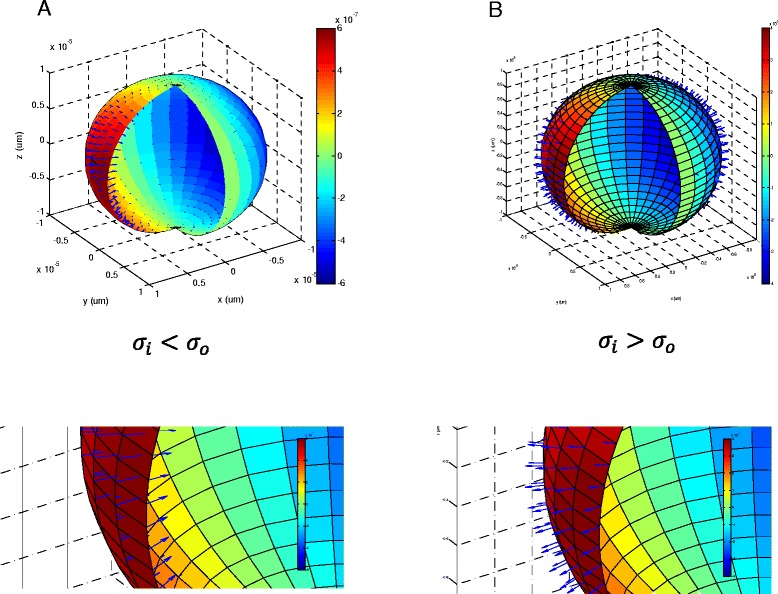
**Distribution of the instant radial pressure on the vesicle surface and its dependency on the media conductivities. A**. Radial pressure compressed the cell if the extracellular conductivity was greater than intracellular conductivity (*σ*_*o*_ = 1.2*S*/*m*, *σ*_*i*_ = 0.3*S*/*m*). **B**. Radial pressure stretched the cell if the intracellular conductivity was greater (*σ*_*i*_ = 1.2*S*/*m. σ*_*o*_ = 0.3*S*/*m*). The color represented the amount of the charge density (C/m^2^) calculated with the parameters in Table 1. Field frequency was 10 KHz.

Equation 24 illustrated the steady part of the surface pressure on the vesicle. The oscillatory part was illustrated in equation (A-7) by the term *e*^*jωt*^ , where *ω* was the angular frequency of the externally applied magnetic field. The vesicle was under the oscillatory pressure with the same frequency of the applied field. Based on equation (24), the maximal pressure was at $\theta =\frac{\pi }{2}, = 0$. In the case of a biological cell, the difference between the dielectric constants of the cytosol and the cell environment could be very small (*ε*_o_ = *ε*_*i*_). Therefore, the sign (direction) of the pressure depends on the conductivity ratio between the cytoplasmic and the extracellular media. Figure 4A plots the case that $\frac{\sigma_i}{\sigma_o}<1,$ when the radial pressure compressed the cell on its equator. Whereas, Figure 4B plots the case that $\frac{\sigma_i}{\sigma_o}>1,$ when the radial pressure stretched the vesicle on its equator.

**Figure 5:**
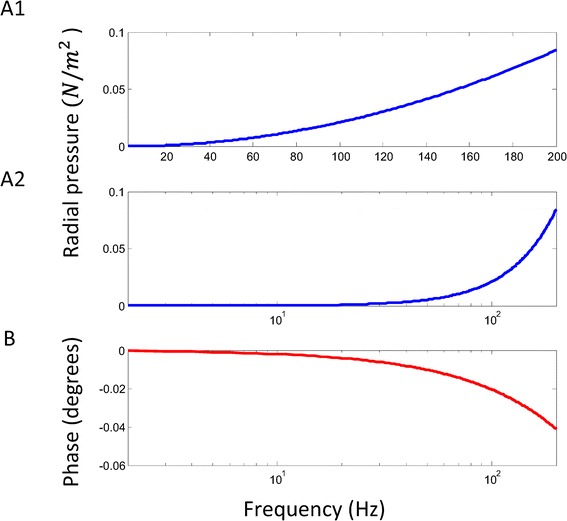
**Frequency dependence of the radial pressure.** Absolute value of the amplitude of the radial pressure over the vesicle was plotted. Notation and parameter values were the same as in Figure 2A. **A**. The overall radial force as a function of the field frequency in a linear plot (A1) and in a log plot (A2). **B**. Phase of the radial force as a function of the field frequency. Phase lag was defined between the phases of the magnetic field and the radial pressure.

Figure 5 plots the frequency dependency of the radial pressure. An increase in the frequency (above 10 KHz) could cause an increase in the magnitude in radial pressure and a slight phase change. At 10 KHz, the maximal radial pressure was 2 × 10^− 4^*N*/*m*^2^, and at 200 KHz, the maximal radial pressure was 8.4 × 10^− 2^*N*/*m*^2^. These calculated pressures are sufficiently smaller in comparison with the ones that are used for mechanical deformation of the cell membrane, including atomic force microscopy, micropipette aspiration, magnetic bead mocrorheology (twisting and pulling), or optical trapping [37].

### Translation forces due to field-charge interactions

After transformation from spherical to rectangular coordinates, we obtained the expressions of the electric field in (*x*, *y*, *z*) directions.

25 $$ \left[\begin{array}{c}\hfill {E}_{ox}\hfill \\ {}\hfill {E}_{oy}\hfill \\ {}\hfill {E}_{oz}\hfill \end{array}\right]=\left[\begin{array}{ccc}\hfill \sin \theta \cos \varphi \hfill & \hfill \cos \theta \cos \varphi \hfill & \hfill - \sin \varphi \hfill \\ {}\hfill \sin \theta \sin \varphi \hfill & \hfill \cos \theta \sin \varphi \hfill & \hfill \cos \varphi \hfill \\ {}\hfill \cos \theta \hfill & \hfill - \sin \theta \hfill & \hfill 0\hfill \end{array}\right]\left[\begin{array}{c}\hfill {E}_{or}\hfill \\ {}\hfill {E}_{o\theta}\hfill \\ {}\hfill {E}_{o\varphi}\hfill \end{array}\right] $$

Immediately outside the vesicle, we found

26 $$ {E}_{ox}=-\frac{j\omega {B}_0C}{2}\left({ \sin}^2\theta { \cos}^2\varphi \frac{3{S}_i}{2{S}_o+{S}_i}+{ \cos}^2\theta { \cos}^2\varphi \frac{3{S}_o}{2{S}_o+{S}_i}+{ \sin}^2\varphi \frac{3{S}_o}{2{S}_o+{S}_i}\right)+\frac{j\omega {B}_0R}{2} \sin \theta \sin \varphi $$

27 $$ {E}_{0y}=-\frac{j\omega {B}_0C}{2}{ \sin}^2\theta \sin \varphi \cos \varphi \frac{3\left({S}_i-{S}_o\right)}{2{S}_o+{S}_i}-\frac{j\omega {B}_0R}{2} \sin \theta \cos \varphi $$

28 $$ {E}_{0z}=-\frac{j\omega {B}_0C}{2}\frac{3\left({S}_i-{S}_o\right)}{2{S}_o+{S}_i} \sin \theta \cos \theta \cos \varphi $$

The overall force that translated the vesicle in the x, y and z directions was:

29 $$ {F}_x={\displaystyle \underset{\theta, \varphi }{\iint }{E}_{ox}{\rho}_sda}=0 $$

30 $$ {F}_y={\displaystyle \underset{\theta, \varphi }{\iint }{E}_{oy}{\rho}_sda}=\frac{\varepsilon_i{S}_o-{\varepsilon}_o{S}_i}{S_i+2{S}_o}{\omega}^2{B}_0^2{R}^3C\pi $$

31 $$ {F}_z={\displaystyle \underset{\theta, \varphi }{\iint }{E}_{oz}{\rho}_sda}=0 $$

**Figure 6:**
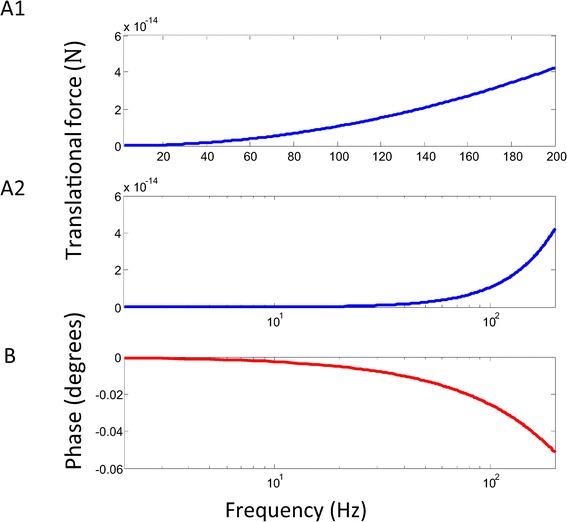
**Frequency dependence of translational force.** Absolute value of the amplitude of the net translational force was plotted. Notation and parameter values were the same as in Figure 2A. **A**. Maximal amplitude of translational force as a function of the field frequency in a linear plot (A1) and in a log plot (A2). **B**. Phase of the translational as a function of the field frequency. Phase lag was defined between the phases of the magnetic field and the translational force.

Therefore, the forces generated by the interaction between the induced surface charges and the field could theoretically contributed to the translational movement of the cell. The force was dependent of both the frequency and conductivity ratios. Figure 6 illustrated the frequency dependency of the translation force. Higher field frequency was associated with larger translational force. However, these forces were quantitatively trivial to introduce vesicle movement. For TMS frequency of 10 KHz, the translational force was 1.1 × 10^−16^*N*. At 200 KHz, the translational force was 4.2 × 10^−14^*N*. A force of 10^−9^*N* to 10^−5^*N* is needed for cell migration to occur in the electric field [37].

*5. Rotation Torques* due to field-charge interactions.

Rotation of the cells could be established by computing the torque on the cell [12] [38]. The tangential $\overset{\rightharpoonup }{\theta }$ and $\overset{\rightharpoonup }{\varphi }$ components of the coulomb forces could potentially generate rotational torques on the vesicle. As illustrated in Appendix D, the overall torque generated by the force in the $\overset{\rightharpoonup }{\theta }$ direction was

32 $$ {\overset{\rightharpoonup }{T}}_{\theta }={\displaystyle \underset{\theta, \varphi }{\iint}\left(R\overset{\rightharpoonup }{R}\right)\times \left({E}_{o\theta }{\rho}_sda\overset{\rightharpoonup }{\theta}\right)} d\theta d\varphi =0 $$

The overall torque generated by the force in the $\overset{\rightharpoonup }{\varphi }$ direction was

33 $$ {\overset{\rightharpoonup }{T}}_{\varphi }={\displaystyle \underset{\theta, \varphi }{\iint}\left(R\overset{\rightharpoonup }{R}\right)\times}\left({E}_{o\varphi }{\rho}_sda\overset{\rightharpoonup }{\varphi}\right) d\theta d\varphi =0 $$

Therefore, the interaction between the induced charges and the field was not likely applying rotational torques.

## Discussion

An analytical, three-dimensional modeling of the interaction of time-varying magnetic fields with cells and vesicles is valuable because it would provide benchmarks for the validation of more general numerical solutions to such problems. Our paper provides the first analytical expressions for surface charges, radial pressure, translational electric forces, and rotational torques generated by a time-varying magnetic field on a model vesicle. The induced surface charge, generated by the inhomogeneity between the vesicle and the medium surrounding the vesicle, interacted with the electric field and produced a biomechanical impact on the vesicle. Four major discoveries were generated from this model. First, distribution of the induced surface charges depended on the orientation of the coil to the vesicle, and the density of these induced charges could be trivial at low frequency range. Secondly, direction of the radial pressure generated on the vesicle depended on the conductivity ratio between the vesicle and the media. Thirdly, the magnetic field could generate translational force but not rotational torque. Lastly, both the compressing pressure and the translational force were trivial at all frequency ranges considered. These data imply a safe clinical application of TMS with its current parameter setup.

### Similarities and differences to electric stimulation

Our analysis of vesicle biomechanics under time-varying magnetic stimulation revealed several commonalities and differences to those under electric stimulation. The build-up of electrical pressure was due to the interaction between the electric field and the induced charges on the vesicle surface. In electric stimulation, this is achieved through directly applied electric current via electrodes. However in magnetic stimulation, electric field is produced by electromagnetic induction. Both mechanisms have been shown to affect cellular physiology, such as causing membrane depolarization [23]. In this paper, we found that the induced surface charge and electric pressure were both dependent on the medium/cytoplasm conductivity ratio, which complies with the results from simulation works in electric stimulation [6,39].

Stimulation of vesicles with time-varying magnetic field is unique in two aspects. First, as a non-invasive method, magnetic stimulation was achieved by current induction inside the tissue, which prevented direct contact with the electrodes and introduces minimal discomfort. Second, the frequency responses of the pressure, force, and torques were different under the two stimulation protocols. In AC electric stimulation, magnitude of the field was constant and independent of its frequency. In magnetic stimulation, however, the magnitude of the induced electric field was proportional to the frequency of the magnetic field (Faraday’s law). Consequently, alternation in the field frequency could affect the vesicle biomechanics in a much more complex manner. As seen, the frequency response of the pressure in the magnetic stimulation (Figure 5) was dramatically different from those proposed for the AC electric field [6,39]. It is unlikely that theories designated for an AC electric field could provide a complete insight to the vesicle biomechanics under a time-varying magnetic field. The following sections will provide more detailed discussion on the biomechanical differences between magnetic stimulation and electric stimulation.

### Surface charges and its frequency dependency

Surface charges and the force generated by the externally applied electric field have been recognized as the basis of cell electrophoresis [40], surface deformation [41] and bending [42]. Furthermore, surface charges have been proposed as the underlying mechanism of fast, cathodal galvanotaxis of rat prostate cancer cells [43]. Here, we show the surface charges (with their density *ρ*_*s*_) were generated on the vesicle under a time-varying magnetic field on the interface of the vesicle and the medium, which represented a boundary of two media with different electrical properties.

Two recent theories have attempted to investigate the surface charge distribution on the vesicle under electric stimulation ([11] for DC, and [6] for AC). Both theories stem from the fact that free charges (ions) could accumulate at the interfaces separating the media with different electrical properties. In both works, the surface charge was a function of the conductivity ratio and the properties of the field. Our analysis on the magnetic field agrees with these conclusions in that the polarity of the charges depends on the conductivity ratio. Moreover, we show that the surface charge distribution depended on both the orientation of the cell to the magnetic coil and the properties of the field (frequency and magnitude). Under magnetic stimulation, the frequency response of the charges was a quasi-linear relationship because surface charge density is dependent on the frequency of the external field.

### Radial forces and its dependency on conductivity ratio and frequency

In addition, we demonstrated that the direction of radial pressure on the vesicle depended on the conductivity ratio between the medium and the cytoplasm (equation 24 and Figure 4), which is compatible with several previous theoretical works on electrodeformation. The dependent nature of the radial pressure applies to several different field types. For DC pulses, the ratio between the conductivities of the inner and outer vesicle solutions dictated the shape deformation induced on lipid vesicles [11]. Sadik et al. [9] found that a strong DC electric field caused giant unilamellar vesicles to prolate elongation along the direction of the electric field when the Intra/extra ratio = 1.92 to 53.0. The similarities between magnetic and electric stimulation could be tested with experimental approaches using these giant vesicles.

The frequency dependency of vesicle deformation under magnetic field stimulation is significantly different from that under AC electric field stimulation. Previous work on electrodeformation indicated the existence of a “critical frequency” around which vesicles switch from elongation to compression [6,7]. Under magnetic field protocol, however, we did not find evidence of this “critical frequency”. Rather, direction of the radial pressure was consistent across large frequency range (Figure 5). Higher field frequency has always implied larger surface pressure. The discrepancy between the magnetic field and the electric field is derived from evidence that magnetic field intensity is proportional to field frequency. This model predication could be tested with giant vesicle experiments under strong, high frequency magnetic field.

### Translational force and rotational torque

An electric force can induce cell migration. Publications regarding this phenomenon can be retrieved as earlier as the 1920s [44], and they have served as a useful tool for the control of cell migration [40,45]. Here, we show that it is possible to generate translational forces with a time-varying magnetic field, with the translational force as non-zero in the y-direction (equation 30, Figure 6), because of the asymmetrical distribution of the induced charges on the vesicle (Figure 2) and the uneven electric field around the vesicle (Eq. 2). However, further analysis revealed these forces to be significantly small in comparison to the force involved in cell migration.

The torque on the cell is related to the orientation of the cells [12,38]. Interestingly, we found the net rotational torques on the vesicle to be zero because torque in the whole vesicle surface was canceled out. It should be noted that both the small translational force and the zero torque computed in this paper are consequences of assuming that force/rotation torque are generated solely by induced surface charges. Theoretically, charged proteins embedded in the cell membrane could also contribute to the translation force and rotation torque [46].

### Implication for the transcranial magnetic stimulation (TMS)

At 10 KHz, a frequency that corresponds with the rising time of the electric pulse used in clinical TMS, electrical compression pressure was insignificant. Under this frequency, the magnetic field generated only 2 × 10^−4^*N*/*m*^2^ of radial pressure. It should be noted that even this value could be a consequence of overestimation in the magnetic field intensity (B_0_), since intensity of the field generated by a coil could decay quickly in the tissue far away from the coil [47]. The duration of the stimulation time was also likely overestimated. During TMS, neuronal responses are induced by pulses as opposed to the mathematically more tractable sinusoidal stimuli used in this model. In this scenario, the magnetically-induced electric field in the tissue (essentially the radial force) is determined by *dB/dt*, which means that the radial force can only be induced during the rise time (and decay time) during a step in the magnetic field. Indeed, rise times of the field affect stimulation in clinical practice with a faster rise time pulse resulting in greater efficiency [48]. Therefore, it is unlikely that the TMS radial force is significant enough to have any physiological implications. To our knowledge and based on a Medline search, there have been no reports on cellular mechanic effects in TMS practice. We do not exclude the possibility that the radial pressure could be large enough to generate compression and become involved in biomechanically-triggered intracellular signaling if much higher field frequencies are implored (i.e., in MHz range).

### Future directions

To simplify the calculation, several assumptions have to be made in this model work. The model did not consider several important biomechanical factors (hydrodynamic force, bending, tension stresses, viscosity and temperature) that may also contribute to vesicle deformation. It also did not explicitly consider the capacitive properties of the membrane compared to fluid-fluid interface, and the voltage across the vesicle surface was assumed continuous. As covered by our analysis, this is valid only if the field frequency is significantly greater than the inverse of the membrane capacitor charging time (above 1–10 KHz). Our previous publication [25] that investigated the “shielding” effect of the cell membrane on the internal organelles indicated that above 1 KHz, the magnetically-induced electric field could start to penetrate into the cell membrane, suggesting a continuous potential distribution across the cell membrane above this frequency. Further work will include the capacitive membrane in the model.

Our paper only investigated induced surface charges where the intrinsic surface charge density was assumed to be zero. While this is likely applicable to the vesicles, since they were usually formed with neutral molecular such as L-a-phosphatidylcholine [7,9,49], biological cells may also contain surface charges such as charged proteins, which reside in the charged lipid headgroups within the membrane itself [46]. Both cationic and anionic functional groups contained in the lipid headgroups contribute to the net electric field at membrane surfaces. The net effect of the relative accumulation of anionic phospholipids in the plasma membrane is an electric field of 10^5^ V/cm, capable of strongly attracting cationic proteins, peptides, and ions [50,51]—a basis for protein targeting and intracellular signaling. It should be noted that the distribution of the surface proteins could be geometrically inhomogeneous, and may also be involved in cell deformation in rare cases. For example, isolated outer hair cells of the cochlea vibrate under the influence of a trans cellular oscillating electric field, which is theoretically due to the interaction between the field and the charged proteins embedded in the cell membrane [52]. In addition, surface charges may undergo a dynamic change in pathological situations. An increased negative surface charge is known to be associated with malignant cancer cells [53,54]. These dynamic changes were shown to affect membrane potential, ion channel distribution, and other cellular activities [55], and should be considered in future studies on cellular biomechanics under magnetic field stimulation.

## Conclusions

We have provided the first analytical solutions for the surface charges, electric field, radial pressure, translational force, and rotation torques of a vesicle under a time-varying magnetic field. The frequency responses of these quantities to the magnetic field were different from that under an AC electric field. At a relative low frequency (10 KHz) similar to that used in clinical TMS, the computed radial pressure, translational forces, and torques on the vesicles are negligible, suggesting that the biological effects of the time-varying magnetic field are not likely caused by alteration of cellular biomechanics.

## Appendix

A Expression of vector potential $\overset{\rightharpoonup }{A}$ in cylindrical coordinates (*r* ', *ϕ* ', *z* ')
  In cylindrical coordinates (*r* ', *ϕ* ', *z* ') centered at *O* ', we had
  A-1 $$ \nabla \times \overset{\rightharpoonup }{A}=\left(\frac{1}{r^{\prime }}\frac{\partial {A}_{z\hbox{'}}}{\partial \phi \hbox{'}}-\frac{\partial {A}_{\phi \hbox{'}}}{\partial z\hbox{'}}\right)\overset{\rightharpoonup }{r}\hbox{'}+\left(\frac{\partial {A}_{r\hbox{'}}}{\partial z\hbox{'}}-\frac{\partial {A}_{z\hbox{'}}}{\partial r\hbox{'}}\right)\overset{\rightharpoonup }{\phi}\hbox{'}+\left[\frac{1}{r\hbox{'}}\frac{\partial }{\partial r\hbox{'}}\left(r\hbox{'}{A}_{\phi \hbox{'}}\right)-\frac{1}{r\hbox{'}}\frac{\partial {A}_{r\hbox{'}}}{\partial \phi \hbox{'}}\right]\overset{\rightharpoonup }{z}\hbox{'} $$
  The magnetic vector potential $\overset{\rightharpoonup }{A}$ induced by an external coil current was
  A-2 $$ \overset{\rightharpoonup }{A}=\frac{\mu }{4\pi }{\displaystyle \iiint \frac{\overset{\rightharpoonup }{J}dv}{R_c}} $$
  where $\overset{\rightharpoonup }{J}$ was the current density in the coil, *R*_*c*_ was the distance between the current element in the coil, and the target tissue where $\overset{\rightharpoonup }{A}$ was evaluated. *dv* was the volume of the element carrying current density $\overset{\rightharpoonup }{J}$, and *μ* was the magnetic conductivity. $\overset{\rightharpoonup }{A}$ was in the direction of $\overset{\rightharpoonup }{\phi}\hbox{'}$ and was symmetrical about the *O* ' *Z* ' axis.
  A-3 $$ {A}_{r\hbox{'}}=0,{A}_{z\hbox{'}}=0,{A}_{\phi \hbox{'}}=A $$
  Substituting (A-3) into (A-1), we had
  A-4 $$ \nabla \times \overset{\rightharpoonup }{A}=\frac{1}{r\hbox{'}}\frac{\partial }{\partial r\hbox{'}}\left(r\hbox{'}{A}_{\phi \hbox{'}}\right)\overset{\rightharpoonup }{z}\hbox{'} $$
  Also:
  A-5 $$ \nabla \times \overset{\rightharpoonup }{A}=\overset{\rightharpoonup }{B}=\overset{\rightharpoonup }{z}\hbox{'}{B}_0{e}^{j\omega t} $$
  From (A-4) and (A-5), we had
  A-6 $$ {A}_{\phi \hbox{'}}=\frac{1}{2}r\hbox{'}{B}_0{e}^{jwt}+\frac{CO}{r\hbox{'}} $$
  where *CO* was a constant. Since the current was symmetrical about the *Z* ' axis. *A*_*ϕ* ' (*r* ' = 0)_ = 0. So *CO* = 0. Therefore,
  A-7 $$ {A}_{\phi \hbox{'}}=\frac{1}{2}r\hbox{'}{B}_0{e}^{j\omega t} $$
B Coordinate transformation from spherical coordinates (*r* ', *θ* ', *ϕ* ') to (*r*, *θ*, *ϕ*) in the computation of the magnetic vector potential $\overset{\rightharpoonup }{A}$
  In spherical coordinates (*r* ', *θ* ', *ϕ* ') with origin at *o* ', from equation (A-7), we had
  B-1 $$ {A}^{\hbox{'}}\left(r\hbox{'}\right)=0 $$
  B-2 $$ {A}^{\hbox{'}}\left(\theta \hbox{'}\right)=0 $$
  B-3 $$ {A}^{\hbox{'}}\left(\phi \hbox{'}\right)=\frac{r\hbox{'}{B}_0}{2} $$
  Here, we have omitted the time factor *e*^*jωt*^ to simplify the calculation, which allowed us to investigate the “instant” effects of the magnetic field on the vesicle. The vector potential was expressed in a Cartesian basis using a matrix transformation,
  B-4 $$ A\hbox{'}\left(x\hbox{'},y\hbox{'},z\hbox{'}\right)=\left[\begin{array}{ccc}\hfill \sin \theta \hbox{'} \cos \phi \hbox{'}\hfill & \hfill \cos \theta \hbox{'} \cos \phi \hbox{'}\hfill & \hfill - \sin \phi \hbox{'}\hfill \\ {}\hfill \sin \theta \hbox{'} \sin \phi \hbox{'}\hfill & \hfill \cos \theta \hbox{'} \sin \phi \hbox{'}\hfill & \hfill \cos \phi \hbox{'}\hfill \\ {}\hfill \cos \theta \hbox{'}\hfill & \hfill \sin \theta \hbox{'}\hfill & \hfill 0\hfill \end{array}\right]\left[\begin{array}{c}\hfill 0\hfill \\ {}\hfill 0\hfill \\ {}\hfill \frac{r\hbox{'}{B}_0}{2}\hfill \end{array}\right]=\left[\begin{array}{c}\hfill -\frac{B_0r\hbox{'} \sin \phi \hbox{'}}{2}\hfill \\ {}\hfill \frac{B_0r\hbox{'} \cos \phi \hbox{'}}{2}\hfill \\ {}\hfill 0\hfill \end{array}\right]=\left[\begin{array}{c}\hfill -\frac{B_0y\hbox{'}}{2}\hfill \\ {}\hfill \frac{B_0x\hbox{'}}{2}\hfill \\ {}\hfill 0\hfill \end{array}\right] $$
  In Figure 1, since *x* ' = *x*, *y* ' = *y* − *c*, *z* ' = *z*, the vector potential $\overset{\rightharpoonup }{A}$ could be expressed in (*x*, *y*, *z*) coordinates as
  B-5 $$ A\left(x,y,z\right)=\left[\begin{array}{c}\hfill -\frac{B_0\left(y-c\right)}{2}\hfill \\ {}\hfill \frac{B_0x}{2}\hfill \\ {}\hfill 0\hfill \end{array}\right] $$
  Expressed in spherical coordinates (*r*, *θ*, *ϕ*),
  B-6 $$ \left[\begin{array}{c}\hfill {A}_{or}\hfill \\ {}\hfill {A}_{o\theta}\hfill \\ {}\hfill {A}_{o\phi}\hfill \end{array}\right]=\left[\begin{array}{ccc}\hfill \sin \theta \cos \phi \hfill & \hfill \sin \theta \sin \phi \hfill & \hfill \cos \theta \hfill \\ {}\hfill \cos \theta \cos \phi \hfill & \hfill \cos \theta \sin \phi \hfill & \hfill - \sin \theta \hfill \\ {}\hfill - \sin \phi \hfill & \hfill \cos \phi \hfill & \hfill 0\hfill \end{array}\right]\left[\begin{array}{c}\hfill -\frac{B_0\left(y-c\right)}{2}\hfill \\ {}\hfill \frac{B_0x}{2}\hfill \\ {}\hfill 0\hfill \end{array}\right]=\left[\begin{array}{c}\hfill \frac{B_0}{2}C \sin \theta \cos \phi \hfill \\ {}\hfill \frac{B_0}{2}C \cos \theta \cos \phi \hfill \\ {}\hfill \frac{B_0}{2}\left(r \sin \theta -C \sin \phi \right)\hfill \end{array}\right] $$
C Determining unknown coefficients *C*_*n*_, *D*_*n*_ in equation (13) using boundary conditions
  Since *V* was bounded at *r* = 0 and *r* → ∞, from equation (13) we had *C*_*o*_ = 0 and *D*_*i*_ = 0. Therefore, expressions for the potential distribution in the extracellular media and in the vesicle cytoplasm were:
  C-1 $$ {V}_o=\frac{D_o}{r^2} \sin \theta \cos \varphi $$
  C-2 $$ {V}_i={C}_ir \sin \theta \cos \varphi $$
  We substituted *A*_0*r*_ (equation 8) and the $\overset{\rightharpoonup }{r}$ components of ∇*V* in the two regions into equation (3) to yield the expressions of the normal components of the electric fields in the two regions:
  C-3 $$ {E}_{or}=-\frac{j\omega {B}_0C}{2} \sin \theta \cos \varphi +\frac{2{D}_o}{r^3} \sin \theta \cos \varphi $$
  C-4 $$ {E}_{ir}=-\frac{j\omega {B}_0C}{2} \sin \theta \cos \varphi -{C}_i \sin \theta \cos \varphi $$
  Following boundary condition (1),
  C-5 $$ \frac{D_o}{R^2}={C}_iR $$
  We then used the boundary condition (2), that the normal components of the current densities were continuous between two different media, to obtain the following equation:
  C-6 $$ {S}_o\left(-\frac{j\omega {B}_0C}{2}+\frac{2{D}_o}{R^3}\right)={S}_i\left(-\frac{j\omega {B}_0C}{2}-{C}_i\right) $$
  We solved (C-5) and (C-6) to obtain the last two coefficients:
  $$ {D}_o=\frac{j\omega {B}_0C}{2}{R}^3\frac{S_o-{S}_i}{2{S}_o+{S}_i} $$
  $$ {C}_i=\frac{j\omega {B}_0C}{2}\frac{S_o-{S}_i}{2{S}_o+{S}_i} $$
D Calculating the rotation torque in equation (32)
  Since $$ \overset{\rightharpoonup }{R}\times \overset{\rightharpoonup }{\theta }=\overset{\rightharpoonup }{\varphi } $$ and $$ \overset{\rightharpoonup }{\varphi }=- \sin \varphi \overset{\frown }{x}+ \cos \varphi \overset{\frown }{y}, $$
  $$ \begin{array}{l}{T}_{\theta }={\displaystyle \underset{\theta, \varphi }{\iint}\left(R\overset{\rightharpoonup }{R}\right)\times \left({E}_{0\theta }q{\rho}_sda\overset{\rightharpoonup }{\theta}\right)} d\theta d\varphi \\ {}={\displaystyle \underset{\theta, \varphi }{\iint }R{E}_{0\theta }q{\rho}_s{R}^2 \sin \theta \overset{\rightharpoonup }{\varphi }} d\theta d\varphi \\ {}={\displaystyle \underset{\theta, \varphi }{\iint }R{E}_{0\theta }q{\rho}_s{R}^2 \sin \theta \left(- \sin \varphi \overset{\frown }{x}+ \cos \varphi \overset{\frown }{y}\right)} d\theta d\varphi \\ {}={\displaystyle \underset{\theta, \varphi }{\iint }{R}^3q\left(-\frac{j\omega {B}_0C}{2}\frac{3{S}_0}{2{S}_0+{S}_2} \cos \theta \cos \varphi \right)\left[-\frac{j\omega {B}_0C}{2}\frac{3\left({\sigma}_2{\varepsilon}_0-{\sigma}_0{\varepsilon}_2\right)}{2{S}_0+{S}_2} \sin \theta \cos \varphi \right] \sin \theta \left(- \sin \varphi \overset{\frown }{x}+ \cos \varphi \overset{\frown }{y}\right)} d\theta d\varphi \\ {}=-{R}^3q{\left(\frac{\omega {B}_0C}{2}\right)}^2\frac{9{S}_0\left({\sigma}_2{\varepsilon}_0-{\sigma}_0{\varepsilon}_2\right)}{{\left(2{S}_0+{S}_2\right)}^2}\left[{\displaystyle \underset{\theta, \varphi }{\iint }{ \sin}^2\theta \cos \theta { \cos}^2\varphi \left(- \sin \varphi \right)\overset{\frown }{x} d\theta d\varphi +{\displaystyle \underset{\theta, \varphi }{\iint }{ \sin}^2\theta \cos \theta { \cos}^2\varphi } \cos \varphi d\theta d\varphi \overset{\frown }{y}}\right]\\ {}=0\end{array} $$

## Abbreviations

*B*_*o*_: Intensity of the time varying magnetic field (Tesla)
*E*: Intensity of the electric field induced by time-varying magnetic field (V/m)
*ρ*_*s*_: Surface density of induced surface charges (C/m2)
*Q*_*s*_: Net induced surface charges (C)
*E*_*ox*_, *E*_*oy*_*E*_*oz*_: Intensity of electric field in the medium (V/m) at $$ \overset{\rightharpoonup }{x}, $$$$ \overset{\rightharpoonup }{y}, $$ and $$ \overset{\rightharpoonup }{z} $$ directions, respectively
*E*_*ix*_, *E*_*iy*_, *E*_*iz*_: Intensity of electric field inside the vesicle (V/m) at $$ \overset{\rightharpoonup }{x}, $$$$ \overset{\rightharpoonup }{y}, $$ and $$ \overset{\rightharpoonup }{z} $$ directions, respectively
*P*_*r*_: Surface pressure (N/ m2)
*F*_*x*_, *F*_*y*_, *F*_*z*_: Translation force (N) applied to the vesicle in $\overset{\rightharpoonup }{x}$, $\overset{\rightharpoonup }{y}$, and $\overset{\rightharpoonup }{z}$ directions, respectively
*T*_*θ*_: *T*_*φ*_, Torques (Nm) generated on the vesicle in $\overset{\rightharpoonup }{\theta }$ and $\overset{\rightharpoonup }{\varphi }$ directions, respectively
